# Comparative Effectiveness and Modality‐Dependent Prognostic Value of Pathological Response Following Neoadjuvant Therapy for Locally Advanced Gastric Cancer

**DOI:** 10.1002/cam4.71619

**Published:** 2026-02-11

**Authors:** Yongfeng Zhu, Zhenchong Chen, Minju Jo, Shoucheng Feng, Yi Zeng, Xiaojiang Chen, Jianrong Guo, Chao Ding, Yukai Jin, Haibo Qiu

**Affiliations:** ^1^ Department of Gastric Surgery, State Key Laboratory of Oncology in South China, Collaborative Innovation Center for Cancer Medicine Sun Yat‐Sen University Cancer Center Guangzhou Guangdong People's Republic of China

**Keywords:** locally advanced gastric cancer, neoadjuvant chemoimmunotherapy, neoadjuvant chemoradiotherapy, neoadjuvant chemotherapy, neoadjuvant treatment modalities, pathology–prognosis mismatch

## Abstract

**Background:**

Neoadjuvant therapies, including chemotherapy (NACT), chemoradiotherapy (NACRT), and chemoimmunotherapy (NACIT), are standard for locally advanced gastric cancer (LAGC). Pathological response is a key surrogate for treatment effectiveness, but its correlation with long‐term outcomes across modalities remains unclear.

**Methods:**

This retrospective cohort study analyzed 256 LAGC patients receiving neoadjuvant therapy (NACT *n* = 162; NACRT *n* = 48; NACIT *n* = 46) from January 2017 to December 2022. Pathological responses, disease‐free survival (DFS), and overall survival (OS) were evaluated using Kaplan–Meier estimates and Cox models.

**Results:**

Compared to NACT, NACIT was associated with significantly improved DFS (HR = 0.75, *p* = 0.035), though no OS difference was observed. Although NACRT enhanced pathological response rates, this was not accompanied by a survival benefit. Crucially, major pathological response (MPR) strongly correlated with improved survival in the NACT and NACIT groups, but no significant association was observed in the NACRT group.

**Conclusion:**

NACIT is associated with improved DFS for patients with LAGC, whereas NACRT suggests a ‘pathology‐prognosis mismatch,’ where improved pathological response may not reliably predict a survival advantage. These findings challenge the uniform application of pathological response as a surrogate endpoint and highlight the critical need for modality‐specific interpretation when evaluating neoadjuvant therapy efficacy.

## 1 Introduction

Gastric cancer (GC) ranks among the most common malignancies of the digestive system worldwide. In China, its incidence is the fifth highest among all cancers, and its mortality rate ranks third [1]. Owing to the insidious onset of early‐stage disease and insufficient public awareness of screening programs, approximately 70% of patients are diagnosed at a locally advanced stage [2, 3, 4]. Neoadjuvant therapy has been validated in multiple studies as an effective strategy to reduce tumor burden, increase the rate of R0 resection, and improve prognosis [5, 6, 7, 8, 9], and it has become an established standard of care for locally advanced gastric cancer (LAGC) [10, 11]. Although neoadjuvant chemotherapy is the most commonly recommended approach in clinical guidelines, some patients demonstrate limited sensitivity to chemotherapy, leading to suboptimal postoperative pathological responses and diminished therapeutic benefit. These limitations underscore the necessity of exploring more effective neoadjuvant therapy strategies.

In this context, neoadjuvant chemoradiotherapy (NACRT) and neoadjuvant chemoimmunotherapy (NACIT) have emerged as alternative or optimization strategies. Compared to chemotherapy alone, NACRT enhances tumor cytotoxicity, thereby improving tumor regression and pathological response rates [7]. For instance, the NEO‐AEGIS study demonstrated that, in patients with esophagogastric junction (EGJ) cancer, NACRT led to superior R0 resection rates, higher pathological complete response (pCR) rates, and more comprehensive tumor downstaging compared to chemotherapy alone [12]. However, NACRT is also associated with a higher incidence of postoperative complications and treatment‐related toxicities. It is important to note that studies evaluating NACRT in non‐EGJ gastric cancer remain limited, and the most favorable outcomes have been observed in EGJ‐targeted trials. As a result, current clinical guidelines do not endorse the routine use of NACRT for non‐EGJ gastric cancer patients. Meanwhile, with the advent of immune checkpoint inhibitors combined with chemotherapy, neoadjuvant chemoimmunotherapy (NACIT) has emerged as a promising alternative. Clinical trials have demonstrated significantly improved pCR rates with NACIT compared to NACT [13, 14, 15]. Nonetheless, immune‐related adverse events may compromise patient tolerance to preoperative therapy and delay surgical scheduling, necessitating careful consideration of the balance between therapeutic effectiveness and safety. Therefore, this study was conducted to evaluate the comparative effectiveness of NACRT and NACIT versus traditional NACT, focusing on both pathological outcomes and survival benefits in a real‐world cohort.

Major pathological response (MPR) and pCR have been widely adopted as surrogate endpoints for assessing neoadjuvant treatment effectiveness and are increasingly recognized as standard markers in clinical practice. However, while pathological response markers such as tumor regression grade (TRG) have demonstrated prognostic value in certain contexts [16, 17, 18], the relationship between these surrogate endpoints and long‐term survival outcomes remains inadequately understood, and their predictive utility across fundamentally different neoadjuvant regimens has not been systematically validated. This raises a critical scientific question regarding whether the association between these short‐term endpoints and long‐term survival is consistent across NACT, NACRT, and NACIT. We hypothesize that the predictive power of MPR/pCR as surrogate endpoints for long‐term prognosis is indeed altered by the specific neoadjuvant regimen administered. Accordingly, this study will investigate the intricate relationship between these pathological response characteristics and long‐term outcomes to assess their suitability as surrogate endpoints in different therapeutic contexts. To ultimately advance the goal of precision therapy, this study will also systematically analyze the key clinicopathological factors influencing disease‐free survival (DFS). The aim is to elucidate the crucial determinants of patient prognosis under different therapeutic backgrounds and pathological response statuses, thereby providing scientific evidence to optimize neoadjuvant therapy selection for LAGC and promote a more individualized approach to treatment.

## 2 Materials and Methods

### 2.1 Study Design and Ethical Approval

This retrospective cohort study enrolled patients diagnosed with LAGC at Sun Yat‐sen University Cancer Center (Guangzhou, China) between January 2017 and December 2022. All patients received neoadjuvant therapy followed by radical gastrectomy. Comprehensive clinical and pathological data were retrieved for analysis. The study protocol was approved by the Ethics Committee of Sun Yat‐sen University Cancer Center (Approval No.: B2025‐146‐01). All data were handled in strict accordance with applicable confidentiality and privacy protection regulations.

### 2.2 Inclusion and Exclusion Criteria

Patients were eligible for inclusion if they met the following criteria: (1) histologically confirmed gastric adenocarcinoma based on endoscopic biopsy; (2) radiologically confirmed locally advanced disease (cT2–T4 and/or cN+, without distant metastasis) as determined by contrast‐enhanced CT, MRI, or PET‐CT; (3) received at least two cycles of neoadjuvant therapy, including chemotherapy, chemoradiotherapy, or chemoimmunotherapy; and (4) underwent R0 resection confirmed by negative margins through D2 or D2+ lymphadenectomy performed at our center following neoadjuvant therapy.

Patients were excluded if they met any of the following criteria: (1) age under 18; (2) history of other malignancies or serious comorbidities; (3) incomplete clinical or pathological data; or (4) loss to follow‐up resulting in outcome data unavailability.

### 2.3 Patient Neoadjuvant Therapy Modalities

**FIGURE 1:**
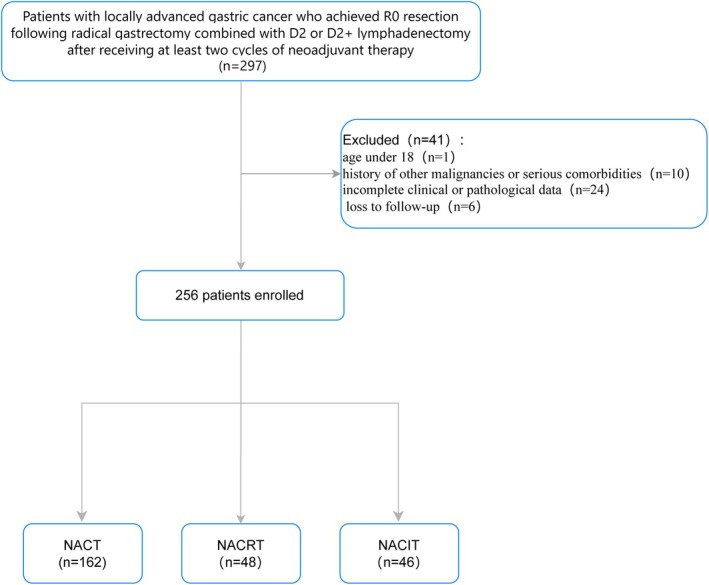
Flowchart of patient selection for the study. NACIT, neoadjuvant chemoimmunotherapy; NACRT, neoadjuvant chemoradiotherapy; NACT, neoadjuvant chemotherapy.

A total of 256 eligible patients were enrolled based on the predefined inclusion and exclusion criteria (Figure 1). Patients were stratified into three groups according to the neoadjuvant therapy modality received: NACT (*n* = 162), NACRT (*n* = 48), and NACIT (*n* = 46). Preoperative treatment plans were determined via multidisciplinary team consensus in accordance with the Chinese Society of Clinical Oncology (CSCO) guidelines for the diagnosis and treatment of gastric cancer.

Patients received 2–6 cycles of chemotherapy, comprising the XELOX regimen (oxaliplatin and capecitabine; *n* = 159), SOX regimen (oxaliplatin and S‐1; *n* = 76), FLOT regimen (5‐fluorouracil, leucovorin, oxaliplatin, and docetaxel; *n* = 12), and FOLFOX regimen (5‐fluorouracil, leucovorin, and oxaliplatin; *n* = 9). Patients in the NACRT group received intensity‐modulated radiotherapy (IMRT) or volumetric‐modulated arc therapy (VMAT), with a total dose of 45 Gy delivered in 25–28 fractions, concurrently with chemotherapy. The NACIT group received chemotherapy in combination with PD‐1 blockade using toripalimab (*n* = 28), nivolumab (*n* = 8), sintilimab (*n* = 8), or tislelizumab (*n* = 2) for 2–8 cycles.

### 2.4 Follow‐Up and Outcome Assessments

Follow‐up began on the first day of neoadjuvant therapy and was maintained through outpatient visits and telephone interviews. Postoperative surveillance consisted of physical examinations, laboratory testing (complete blood counts, liver and renal function panels, and tumor markers), and radiological assessments (CT or MRI). Patients were generally advised to attend follow‐up every 3–6 months during the first 2 postoperative years, then every 6–12 months until 5 years after surgery. Recurrence and survival endpoints were determined based on medical records and follow‐up results documented at our center. Follow‐up continued until documented recurrence, death, loss to follow‐up, or the data cutoff date (December 2024).

The primary study endpoint was DFS, defined as the interval from the date of surgery to the first documented recurrence, distant metastasis, or all‐cause mortality. The secondary endpoint was overall survival (OS), measured from the initiation of neoadjuvant therapy to the date of death or last follow‐up. Pathological treatment response was evaluated strictly according to the tumor regression grading (TRG) system recommended by the National Comprehensive Cancer Network (NCCN) Guidelines for Gastric Cancer. The applied TRG system categorizes response as follows: TRG 0 (complete response), defined as no viable cancer cells in the primary tumor or lymph nodes; TRG 1 (near complete response), defined as single cells or rare small groups of residual cancer cells; TRG 2 (partial response), defined as residual cancer cells with evident tumor regression but more than single cells or rare small clusters; and TRG 3 (poor or no response), defined as extensive residual cancer with no evident tumor regression. Acellular mucin pools that may be present after neoadjuvant therapy were not interpreted as residual viable tumor. All pathological evaluations were performed according to standardized histopathological criteria and underwent a three‐level review process mandated by our center. For analytical purposes, pCR was defined as TRG 0; MPR as TRG 0/1; and nonmajor pathological response (NMPR) as TRG 2/3.

### 2.5 Statistical Analysis

Statistical analyses were conducted using R software (version 4.2.2). Categorical variables were compared using Pearson's *χ* ^2^ test or Fisher's exact test, as appropriate. The Kaplan–Meier method was employed to estimate DFS and OS, and differences between groups were assessed using the log‐rank test. Univariate Cox regression analysis was used to screen for variables associated with DFS. To strictly control for potential confounders and selection bias inherent to the retrospective design, variables with a *p*‐value < 0.05 in univariate analysis were included in a multivariate Cox proportional hazards model to identify independent prognostic factors. A stepwise forward selection procedure was applied for model construction. Statistical significance was set at a two‐sided *p*‐value < 0.05.

## 3 Result

Baseline characteristics of the 256 enrolled patients are summarized in Table 1. No statistically significant differences were observed for any baseline variable (all *p* > 0.05), confirming balanced baseline characteristics among the groups. The age of enrolled patients ranged from 24 to 84 years, with the majority being older than the median age of 61, and most were male. Based on BMI, a higher proportion of overweight or obese patients was observed in the NACRT and NACIT groups, while the NACT group included relatively more underweight individuals. The incidence of hypoalbuminemia and anemia was slightly higher in the NACT group compared to the other two groups. Tumor location varied across the groups: upper gastric tumors predominated in both the NACT and NACRT groups, with over half of the NACRT cases located in the upper stomach, whereas the NACIT group showed a more balanced distribution across upper, middle, and lower regions. Among clinical T stages, T4 was the most prevalent in all groups, with the NACIT group having the highest proportion. N2 disease was the most common clinical N stage across all groups. Histologically, the NACIT group had the highest frequency of poorly differentiated tumors. The presence of signet‐ring cell features was comparable among groups. Regarding Lauren classification, the diffuse subtype was more prevalent in the NACIT group, whereas the intestinal subtype was predominant in the NACT and NACRT groups.

**TABLE 1:** Baseline characteristics of patients with locally advanced gastric cancer across neoadjuvant treatment groups.

Characteristic	NACT	NACRT	NACIT	*p*
Age (%)				
≤ 61	77 (47.5)	26 (54.2)	19 (41.3)	0.458
> 61	85 (52.5)	22 (45.8)	27 (58.7)	
Sex (%)				
Male	119 (73.5)	34 (70.8)	27 (58.7)	0.154
Female	43 (26.5)	14 (29.2)	19 (41.3)	
BMI (%)				
Underweight	27 (16.7)	4 (8.3)	6 (13.0)	0.11
Normal weight	96 (59.3)	25 (52.1)	22 (47.8)	
Overweight/Obese	39 (24.1)	19 (39.6)	18 (39.1)	
Hypoproteinemia (%)				
No	66 (40.7)	23 (47.9)	21 (45.7)	0.624
Yes	96 (59.3)	25 (52.1)	25 (54.3)	
Anemia (%)				
No	99 (61.1)	33 (68.8)	31 (67.4)	0.53
Yes	63 (38.9)	15 (31.2)	15 (32.6)	
Site (%)				
Upper	77 (47.5)	26 (54.2)	15 (32.6)	0.272
Middle	42 (25.9)	12 (25.0)	17 (37.0)	
Lower	43 (26.5)	10 (20.8)	14 (30.4)	
Clinical T stage (%)				
T3	56 (34.6)	12 (25.0)	11 (23.9)	0.24
T4	106 (65.4)	36 (75.0)	35 (76.1)	
Clinical N stage (%)				
N1	8 (4.9)	6 (12.5)	3 (6.5)	0.446
N2	86 (53.1)	24 (50.0)	26 (56.5)	
N3	68 (42.0)	18 (37.5)	17 (37.0)	
Grade (%)				
Poorly differentiated	79 (48.8)	21 (43.8)	27 (58.7)	0.094
Moderately to poorly differentiated	40 (24.7)	12 (25.0)	15 (32.6)	
Moderately differentiated	43 (26.5)	15 (31.2)	4 (8.7)	
SignetRing cells features (%)				
No	134 (82.7)	39 (81.2)	37 (80.4)	0.927
Yes	28 (17.3)	9 (18.8)	9 (19.6)	
Lauren classification (%)				
Intestinal	78 (48.1)	24 (50.0)	15 (32.6)	0.188
Diffuse	44 (27.2)	10 (20.8)	19 (41.3)	
Mixed	40 (24.7)	14 (29.2)	12 (26.1)	

Abbreviations: NACIT, neoadjuvant chemoimmunotherapy; NACRT, neoadjuvant chemoradiotherapy; NACT, neoadjuvant chemotherapy.

Postoperative clinical and pathological characteristics of the three treatment groups are summarized in Table 2. The proportions of patients receiving adjuvant therapy were comparable across the NACT, NACRT, and NACIT groups (*p* = 0.731). However, significant differences were observed in pathological responses. Both the NACRT and NACIT groups exhibited markedly higher rates of major pathological response (MPR) and a greater proportion of patients with lower pathological T stages (ypT0–2) compared to the NACT group. Notably, the NACRT group exhibited the greatest proportion of ypN0–1 nodal status, along with the lowest rates of lymphovascular invasion and perineural invasion, in comparison to the other two groups. These findings suggest a superior local tumor regression effect in the NACRT cohort.

**TABLE 2:** Postoperative clinicopathological characteristics of patients with locally advanced gastric cancer across neoadjuvant treatment groups.

Characteristic	NACT	NACRT	NACIT	*p*
Adjuvant therapy				
No	24 (14.8)	5 (10.4)	6 (13.0)	0.731
Yes	138 (85.2)	43 (89.6)	40 (87.0)	
MPR				
No	127 (78.4)	25 (52.1)	26 (56.5)	< 0.001
Yes	35 (21.6)	23 (47.9)	20 (43.5)	
ypT (%)				
T0–2	35 (21.6)	27 (56.2)	26 (56.5)	< 0.001
T3–4	127 (78.4)	21 (43.8)	20 (43.5)	
ypN (%)				
N0–N1	79 (48.8)	36 (75.0)	26 (56.5)	0.006
N2–N3	83 (51.2)	12 (25.0)	20 (43.5)	
LVI (%)				
No	99 (61.1)	40 (83.3)	34 (73.9)	0.009
Yes	63 (38.9)	8 (16.7)	12 (26.1)	
PNI (%)				
No	76 (46.9)	42 (87.5)	27 (58.7)	< 0.001
Yes	86 (53.1)	6 (12.5)	19 (41.3)	

Abbreviations: LVI, lymphovascular invasion; MPR, major pathology response; NACIT, neoadjuvant chemoimmunotherapy; NACRT, neoadjuvant chemoradiotherapy; NACT, neoadjuvant chemotherapy; PNI, perineural invasion.

The median follow‐up duration for the entire cohort of 256 patients was 43 months (range: 11.5–93 months). The median disease‐free survival (DFS) and overall survival (OS) for each treatment group were as follows: in the NACT group, the median DFS was 25 months (95% CI: 16–42 months), while the median OS was not reached (NR) by the time of the final follow‐up. In the NACRT group, the median DFS was 28 months (95% CI: 18 months to upper limit not assessable at the time of analysis), with the median OS also remaining unreached. Similarly, in the NACIT group, both the median DFS and OS were not reached by the time of the final follow‐up.

**FIGURE 2:**
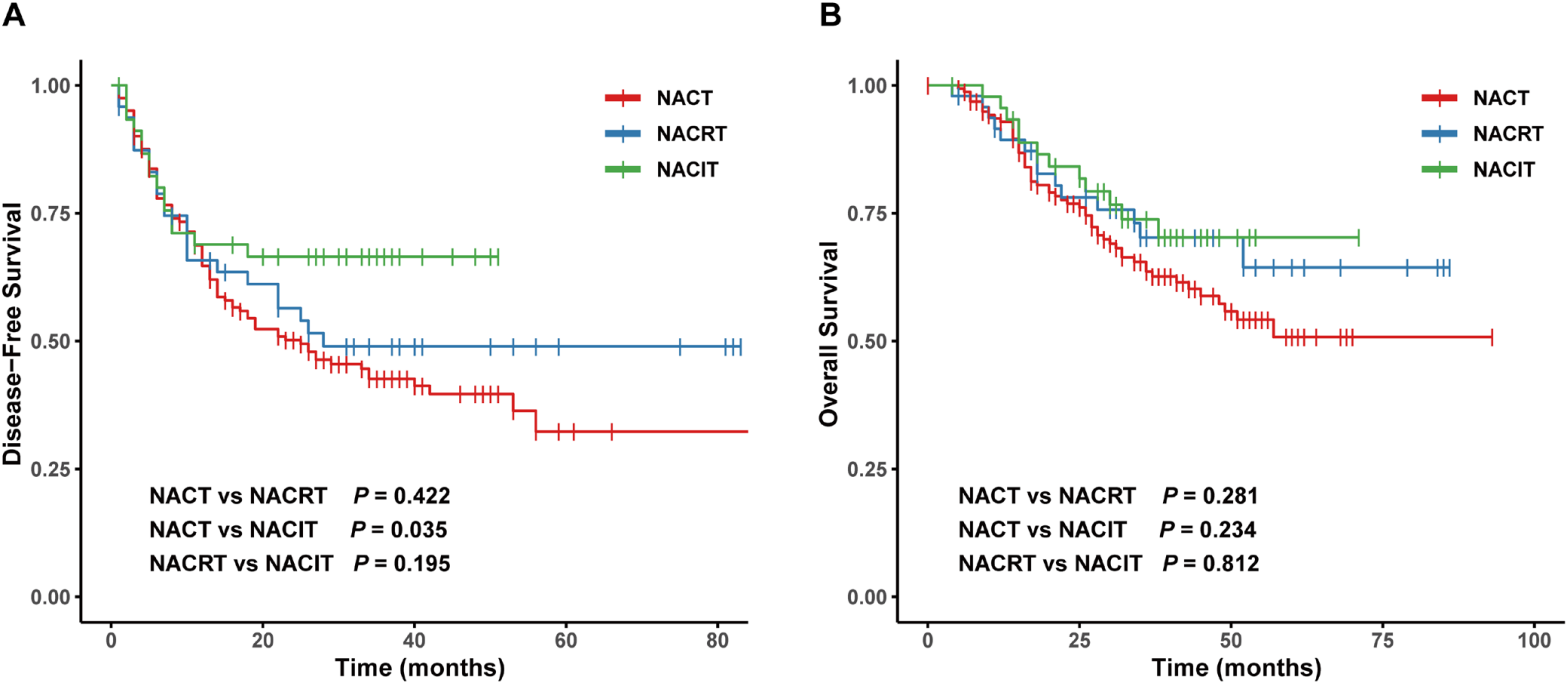
Kaplan–Meier survival curves for disease‐free survival (DFS) and overall survival (OS) among patients receiving different neoadjuvant therapies (NACT, NACRT, and NACIT). (A) DFS for patients undergoing NACT, NACRT, and NACIT. (B) OS for patients undergoing NACT, NACRT, and NACIT. Pairwise comparison *p*‐values are displayed in the bottom‐left corner of each panel. DFS, disease‐free survival; NACIT, neoadjuvant chemoimmunotherapy; NACRT, neoadjuvant chemoradiotherapy; NACT, neoadjuvant chemotherapy; OS, overall survival.

As illustrated in Figure 2, no statistically significant differences in DFS or OS were detected between the NACRT and NACT groups (DFS: HR = 0.83, 95% CI: 0.53–1.32, *p* = 0.422; OS: HR = 0.73, 95% CI: 0.40–1.30, *p* = 0.281). Compared to the NACT group, the NACIT group was associated with significantly longer DFS (HR = 0.75, 95% CI: 0.57–0.98, *p* = 0.035), although the difference in OS was not statistically significant (HR = 0.83, 95% CI: 0.61–1.13, *p* = 0.234). No significant differences in either DFS or OS were observed between the NACRT and NACIT groups (DFS: HR = 0.65, 95% CI: 0.34–1.25, *p* = 0.195; OS: HR = 0.91, 95% CI: 0.42–1.98, *p* = 0.812).

**FIGURE 3:**
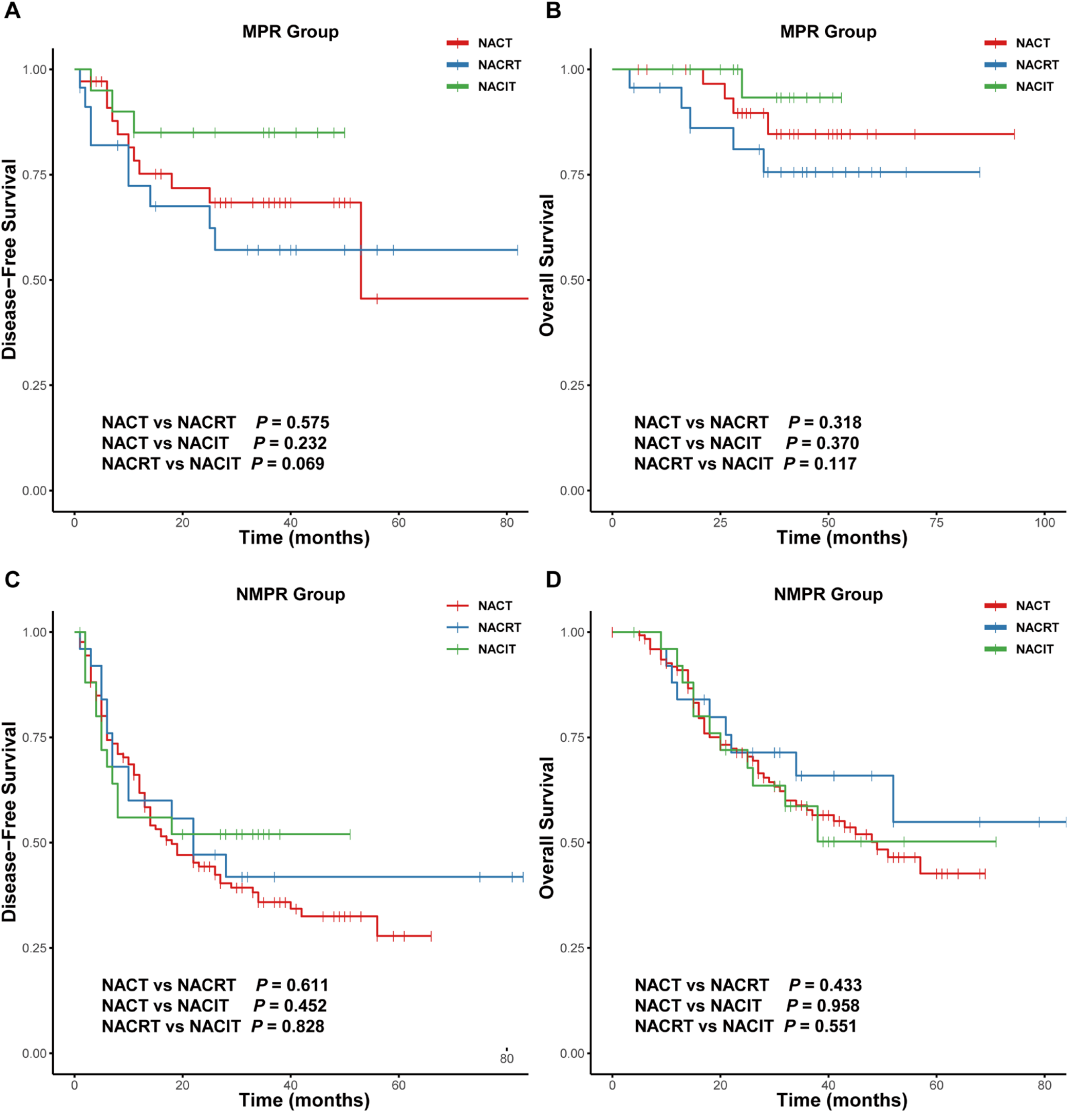
Kaplan–Meier survival curves for disease‐free survival (DFS) and overall survival (OS) in the MPR and NMPR subgroups based on neoadjuvant treatment modalities (NACT, NACRT, and NACIT). (A, B) DFS and OS for the MPR group stratified by NACT, NACRT, and NACIT. (C, D) DFS and OS for the NMPR group stratified by NACT, NACRT, and NACIT. Pairwise comparison *p*‐values are displayed in the bottom‐left corner of each panel. DFS, disease‐free survival; MPR, major pathological response; NACIT, neoadjuvant chemoimmunotherapy; NACRT, neoadjuvant chemoradiotherapy; NACT, neoadjuvant chemotherapy; NMPR, no major pathological response; OS, overall survival.

Patients were further stratified into MPR and NMPR subgroups according to postoperative tumor regression status. Figure 3 illustrates DFS and OS outcomes across these subgroups for each neoadjuvant therapy modality. In the MPR subgroup, no statistically significant pairwise differences in DFS or OS were observed among the NACT, NACRT, and NACIT groups (all *p* > 0.05). However, a nonsignificant trend toward improved DFS was noted in the NACIT group compared to the NACRT group (HR = 0.32, 95% CI: 0.09–1.17, *p* = 0.069). Likewise, in the NMPR subgroup, no significant differences in DFS or OS were detected across the three treatment groups (all *p* > 0.05).

**FIGURE 4:**
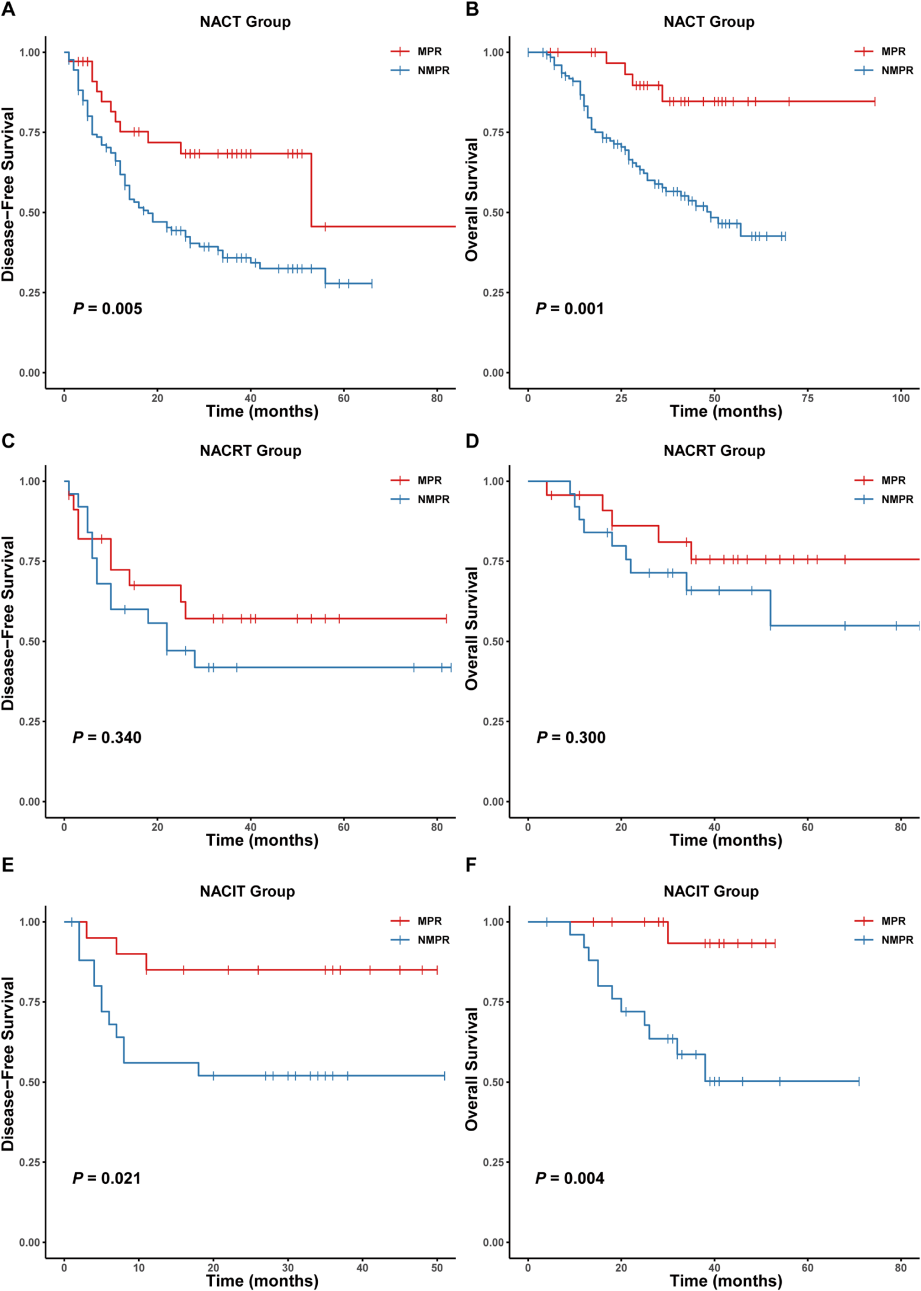
Kaplan–Meier survival curves for disease‐free survival (DFS) and overall survival (OS) stratified by pathological response (MPR vs. NMPR) in each neoadjuvant therapy group. (A, B) DFS and OS for patients in the NACT group. (C, D) DFS and OS for patients in the NACRT group. (E, F) DFS and OS for patients in the NACIT group. DFS, disease‐free survival; MPR, major pathological response; NACIT, neoadjuvant chemoimmunotherapy; NACRT, neoadjuvant chemoradiotherapy; NACT, neoadjuvant chemotherapy; NMPR, no major pathological response; OS, overall survival.

To further assess the prognostic significance of MPR, patients were stratified according to the neoadjuvant therapy modality, and survival outcomes were analyzed (Figure 4). In the NACT group, achieving MPR was associated with significantly improved DFS and OS compared to those with NMPR (DFS: HR = 2.38, 95% CI: 1.27–4.48, *p* = 0.005; OS: HR = 4.62, 95% CI: 1.67–12.75, *p* = 0.001). In contrast, among patients in the NACRT group, no statistically significant association was observed between pathological response and survival outcomes (DFS: HR = 1.495, 95% CI: 0.646–3.461, *p* = 0.340; OS: HR = 1.772, 95% CI: 0.592–5.298, *p* = 0.300). In the NACIT group, achieving MPR was associated with significantly improved prognosis compared to NMPR, with significant survival advantages in both DFS (HR = 4.013, 95% CI: 1.130–14.248, *p* = 0.021) and OS (HR = 11.258, 95% CI: 1.444–87.738, *p* = 0.004).

**FIGURE 5:**
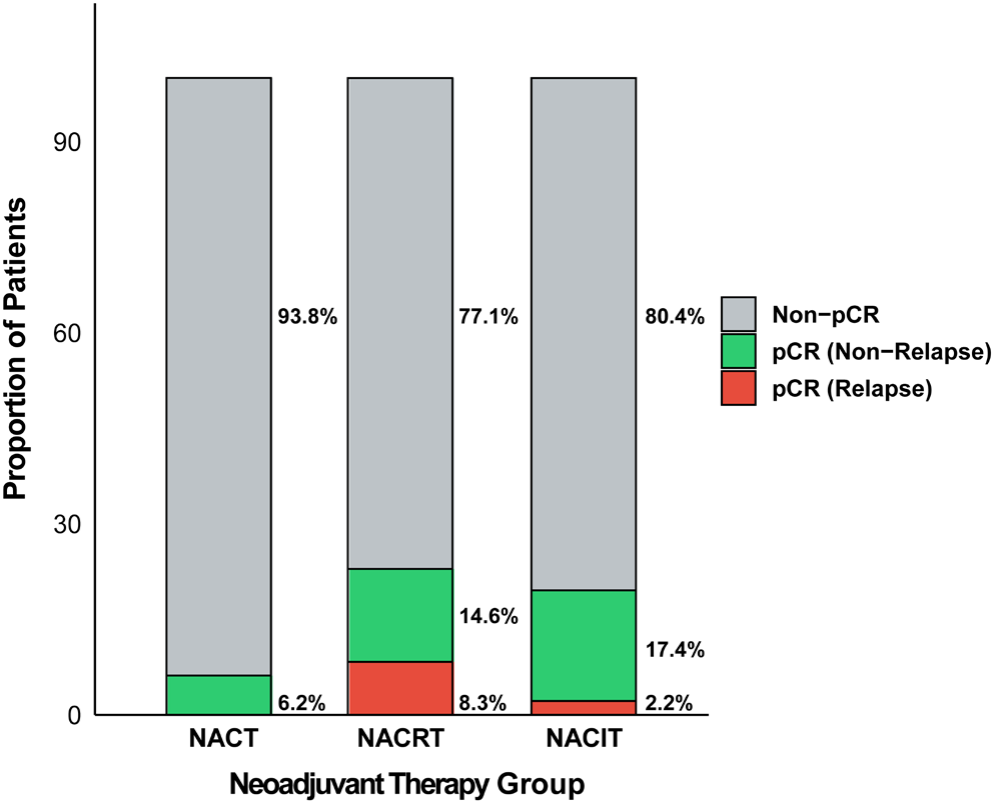
Pathological complete response (pCR) rates and recurrence status among pCR patients in each treatment group. Gray: Proportion of non‐pCR patients. Green: Proportion of patients who achieved pCR without recurrence. Red: Proportion of patients who achieved pCR but experienced recurrence. Corresponding proportions are labeled on the right. NACIT, neoadjuvant chemoimmunotherapy; NACRT, neoadjuvant chemoradiotherapy; NACT, neoadjuvant chemotherapy.

Figure 5 presents the pCR rates and recurrence outcomes among pCR patients across the three groups. A total of 30 patients achieved pCR: the highest pCR rate was observed in the NACRT group (31.2%), followed by the NACIT group (19.6%), and the NACT group (6.2%). Among patients who achieved pCR, recurrence rates differed substantially. The NACRT group exhibited the highest recurrence rate at 36.4%, whereas the NACIT group had a recurrence rate of 11.1%. Notably, no recurrence was observed in pCR patients from the NACT group.

Cox proportional hazards regression was conducted to identify independent predictors of DFS, as summarized in Table 3. On univariate analysis, multiple clinical and pathological variables were significantly associated with DFS, including age, type of neoadjuvant therapy, tumor differentiation, signet‐ring cell histology, Lauren classification, MPR status, ypT stage, ypN stage, lymphovascular invasion (LVI), and perineural invasion (PNI) (all *p* < 0.05). Adjuvant therapy was not significantly associated with DFS (*p* = 0.265) and was therefore not included in the multivariate model. On multivariate analysis, age > 61 years, ypN stage, and PNI were identified as independent prognostic factors associated with DFS (all *p* < 0.05).

**TABLE 3:** Univariate and multivariate analyses of risk factors potentially associated with disease‐free survival in patients with locally advanced gastric cancer across neoadjuvant treatment groups.

Factor	Univariate analysis		Multivariate analysis	
	HR (95% CI)	*p*	HR (95% CI)	*p*
Age				
≤ 61				
> 61	0.57 (0.40–0.82)	0.002	0.59 (0.40–0.88)	0.009
Neoadjuvant therapy				
NACT				
NACRT	0.83 (0.53–1.32)	0.440	1.29 (0.79–2.11)	0.303
NACIT	0.55 (0.32–0.96)	0.034	0.65 (0.36–1.18)	0.156
Adjuvant therapy				
No				
Yes	0.76 (0.47–1.23)	0.265		
Site				
Upper				
Middle	0.94 (0.62–1.43)	0.768		
Lower	0.88 (0.57–1.36)	0.566		
Grade				
Poorly differentiated				
Moderately to poorly differentiated	0.73 (0.48–1.11)	0.145	0.72 (0.43–1.21)	0.215
Moderately differentiated	0.47 (0.29–0.76)	0.002	0.56 (0.30–1.04)	0.067
SignetRing cells features				
No				
Yes	2.03 (1.36–3.02)	< 0.001	1.19 (0.74–1.91)	0.465
Lauren classification				
Intestinal				
Diffuse	1.83 (1.21–2.78)	0.004	0.75 (0.39–1.43)	0.383
Mixed	1.76 (1.15–2.71)	0.010	1.05 (0.62–1.76)	0.862
MPR				
Yes				
No	0.41 (0.26–0.65)	< 0.001	0.75 (0.43–1.31)	0.307
ypT				
T0–T2				
T3–T4	2.64 (1.71–4.07)	< 0.001	1.32 (0.73–2.39)	0.354
ypN				
N0–N1				
N1–N2	2.68 (1.87–3.84)	< 0.001	1.82 (1.18–2.79)	0.006
LVI				
No				
Yes	1.86 (1.30–2.65)	< 0.001	0.98 (0.66–1.47)	0.941
PNI				
No				
Yes	2.73 (1.91–3.91)	< 0.001	1.64 (1.03–2.61)	0.036

Abbreviations: LVI, lymphovascular invasion; MPR, major pathologic response; NACIT, neoadjuvant chemoimmunotherapy; NACRT, neoadjuvant chemoradiotherapy; NACT, neoadjuvant chemotherapy; NMPR, nonmajor pathologic response; PNI, perineural invasion.

## 4 Discussion

Our findings indicate that NACIT is associated with a significant DFS advantage over NACT in patients with LAGC. This result aligns with recent evidence from the phase III KEYNOTE‐585 trial, which demonstrated that adding pembrolizumab to chemotherapy significantly improved 2‐year event‐free survival (EFS) compared to chemotherapy alone [13]. While the median EFS was not reached at the time of the interim analysis, these findings underscore the potential of NACIT in delaying disease progression and recurrence. Mechanistically, the observed clinical benefit may be explained by the synergistic interaction between chemotherapy and immune checkpoint blockade [19], as proposed by Topalian and colleagues. Specifically, chemotherapy induces immunogenic cell death, leading to the release of tumor antigens, while immunotherapy enhances the capacity of effector T cells to recognize, expand, and eliminate tumor antigens and micrometastatic lesions. However, despite the marked DFS advantage observed in the NACIT group, no statistically significant OS benefit was detected. The absence of a statistically significant OS benefit in the NACIT group, despite its marked DFS advantage, may be explained by several possible factors. One potential explanation is the limited follow‐up duration, which might not have been long enough to fully capture the delayed survival benefit characteristic of immunotherapy. Additionally, variability in postoperative adjuvant regimens among patients likely contributed to the difficulty in making direct group comparisons. Moreover, immune‐related adverse events, including treatment delays, early discontinuation, or, in severe cases, fatal toxicity, may have undermined the long‐term effectiveness of NACIT therapy.

Our findings further reinforce the prognostic significance of TRG in patients with LAGC treated with NACT or NACIT. Specifically, achieving MPR (TRG 0/1) was significantly associated with prolonged DFS and OS, consistent with the results of Abboretti et al., whose retrospective analysis of resectable gastric cancer demonstrated that higher degrees of tumor regression were associated with longer DFS [20], highlighting the utility of TRG as a prognostic biomarker in the neoadjuvant setting. Similarly, Wang et al. provided additional support for the clinical relevance of TRG [21]. Notably, we observed more pronounced tumor regression in the NACIT group compared to the NACT group—a trend that has been consistently validated by multiple clinical trials evaluating the effectiveness of NACIT [13, 14, 15]. These observations suggest that NACIT is associated with higher pathological response rates and has the potential to yield better long‐term outcomes, with TRG remaining a robust prognostic tool in both treatment contexts.

With respect to NACRT, our findings indicated a numerical improvement in both DFS and OS compared to NACT; however, these differences did not reach statistical significance (*p* > 0.05). Although NACRT significantly increased MPR rates relative to NACT, this enhancement in pathological response did not correspond to meaningful improvements in survival outcomes. These results suggest that while MPR remains a valuable measure of short‐term treatment effectiveness, its predictive power for long‐term prognosis may be limited in the context of NACRT.

This phenomenon, termed “pathology–prognosis mismatch,” has been previously reported in multiple trials. The TOPGEAR trial, for instance, demonstrated that NACRT improved pCR and MPR rates and facilitated tumor downstaging, yet failed to yield survival benefits in resectable gastric and gastroesophageal junction adenocarcinomas [22]. Similar findings were observed in the POET [23] and Neo‐AEGIS [12] studies. Several mechanisms may explain this mismatch. One possible explanation is that the pathological response in NACRT may be driven by intense local cytotoxicity rather than serving as a marker of favorable systemic tumor biology, thus leaving micrometastases unaddressed and potentially leading to later distant recurrence [24]. Additionally, the added toxicity burden from radiotherapy may compromise treatment adherence, reduce completion rates, and negatively impact quality of life, thereby counteracting potential survival benefits [25, 26, 27]. Crucially, it is important to acknowledge that chemoradiotherapy often induces distinct histological changes, such as extensive stromal fibrosis and inflammation, which differ fundamentally from chemotherapy‐induced regression. This distinct histological landscape can complicate pathological assessment; for instance, residual micrometastases may be encased in dense fibrotic tissue, potentially escaping detection or remaining dormant only to cause later relapse. Furthermore, favorable pathological responses may result in the reduction, delay, or omission of postoperative adjuvant therapy [22, 24], potentially diminishing the overall effectiveness. It is also important to note that, in accordance with Chinese Society of Clinical Oncology (CSCO) guidelines, NACRT is primarily recommended for cT3–4aN + M0 gastroesophageal junction cancers. In our study, however, the NACRT cohort included patients with nongastroesophageal junction gastric cancer, potentially confounding the observed survival outcomes. Collectively, these findings underscore the unique characteristics and limitations of NACRT in the treatment of LAGC. A comprehensive evaluation framework encompassing both short‐term pathological response and long‐term survival is warranted. Future studies should prioritize optimized radiation strategies, improved integration with systemic therapies, and refined patient selection to identify subpopulations most likely to benefit from NACRT.

In the subgroup analyses of patients with MPR and NMPR, no statistically significant differences in DFS or OS were observed across neoadjuvant therapy modalities (all *p* > 0.05). Achieving MPR appears to be a dominant prognostic factor, as survival outcomes within this subgroup remained consistently favorable, minimizing variability between treatment groups. In the NMPR subgroup, persistent tumor burden and an elevated risk of relapse likely diminished the therapeutic effectiveness across all regimens. The limited sample size and low event frequency within subgroups may have further reduced the ability to detect intergroup differences. Importantly, among MPR patients, the NACIT group demonstrated a trend toward improved DFS compared to NACRT, suggesting that significant tumor regression may enhance the benefits of immunotherapy. This observation aligns with the hypothesis that pathological response may serve more as a marker of favorable underlying tumor biology (e.g., chemosensitivity) rather than solely as a predictor of survival benefit derived from local tumor destruction. Thus, simply intensifying local therapy (as in NACRT) to achieve regression may not necessarily confer the same systemic survival advantage. For NMPR patients, attention should be directed toward optimizing adjuvant therapies to improve effectiveness while minimizing adverse effects. Conversely, in MPR patients, maintenance strategies to mitigate late relapse warrant further exploration.

In this study, the analysis of pCR indicated that, given the observed “pathology–prognosis mismatch,” pCR and MPR may not be reliable surrogate endpoints for predicting the long‐term outcomes of NACRT. In contrast, NACIT not only achieved a relatively favorable pCR rate but also showed a lower recurrence risk among pCR patients, suggesting potential advantages in local control and recurrence prevention. In the NACT group, although no recurrence was observed among pCR patients, the limited number of cases restricted the interpretability of its prognostic significance. Overall, the findings related to pCR in this study are essentially descriptive, and further validation through studies with larger sample sizes is needed in the future.

Univariate and multivariate Cox regression analyses identified age > 61 years as an independent favorable prognostic factor for DFS, which differs from some previous studies. For example, Wang et al. [28] and Brown et al. [29] associated older age with worse outcomes due to immunosenescence, malnutrition, and reduced treatment adherence. Conversely, Liu et al. observed more aggressive tumor biology in younger patients with early‐onset gastric cancer, aligning with our findings [30]. These results suggest that the prognostic role of age may depend on tumor biology and patient‐specific factors. Similarly, PNI positivity was found to be associated with early recurrence and significantly worse outcomes, consistent with previous studies [31, 32, 33]. Notably, the NCCN gastric cancer guidelines explicitly identify younger age (< 50 years) and PNI positivity as key high‐risk factors for gastric cancer recurrence [10], corroborating the importance of these variables in risk stratification. The critical prognostic value of lymph node metastasis was also reflected in our study. Patients with ypN2‐3 staging had a significantly higher risk of recurrence compared to those with ypN0‐1 staging. This observation aligns with the consensus in the guidelines [10, 11] regarding the importance of lymph node staging in assessing the likelihood of gastric cancer recurrence. These findings reinforce the essential role of lymph node involvement as a predictive marker for disease progression and recurrence.

This study has several limitations inherent to its retrospective design. Most importantly, treatment allocation was not randomized, which introduces a significant risk of selection bias due to unmeasured confounding factors that may have influenced both regimen choice and outcomes. The study is further constrained by a modest overall sample size and an imbalanced distribution of patients across the groups, which may have provided insufficient statistical power to detect more subtle survival differences. Additionally, the lack of complete uniformity in chemotherapy regimens, durations, and undocumented treatment‐related toxicities hindered a full safety and efficacy analysis. The follow‐up period was relatively short, particularly for the NACIT cohort, potentially underestimating long‐term benefits. This was compounded by variability in follow‐up intervals among patients, which could introduce bias into the survival analysis. Furthermore, pathological response was analyzed as a categorical variable due to the unavailability of precise residual tumor percentages in retrospective records. We also acknowledge that simplifying the TRG system into a binary MPR category, while necessary to ensure statistical power given the sample size, inevitably limits the assessment of more subtle differences between intermediate response groups. Finally, the absence of molecular biomarker data, such as PD‐L1 expression or MSI status, precluded the exploration of predictive factors for personalized therapy. Therefore, future large‐scale, multicenter prospective studies, ideally with randomization, are warranted to validate these findings, mitigate these biases, and integrate biomarker analyses to ultimately refine and optimize individualized treatment strategies for LAGC.

## 5 Conclusion

In conclusion, this study suggests that NACIT is associated with improved disease‐free survival in patients with LAGC compared to traditional NACT. In stark contrast, while NACRT also enhances pathological response rates, this benefit was not accompanied by improved long‐term survival, highlighting a significant “pathology‐prognosis mismatch” for this modality. Crucially, our findings challenge the uniform application of pathological response as a surrogate endpoint, revealing that its prognostic value appears robust for NACT and NACIT but warrants caution in the context of NACRT. These results underscore the need for modality‐specific evaluation of treatment efficacy and emphasize that long‐term survival data remain indispensable for guiding the optimal neoadjuvant strategy for patients with LAGC.

## Author Contributions

**Yongfeng Zhu:** data curation (lead), formal analysis (lead), methodology (equal), validation (lead), writing – original draft (lead), writing – review and editing (equal). **Zhenchong Chen:** data curation (equal), formal analysis (supporting), methodology (equal), validation (supporting), writing – original draft (supporting), writing – review and editing (equal). **Minju Jo:** data curation (supporting), formal analysis (supporting), methodology (equal), validation (supporting), writing – original draft (supporting), writing – review and editing (supporting). **Shoucheng Feng:** data curation (supporting), formal analysis (supporting), investigation (supporting), writing – review and editing (supporting). **Yi Zeng:** data curation (supporting), formal analysis (supporting), investigation (equal), writing – review and editing (supporting). **Xiaojiang Chen:** data curation (equal), formal analysis (equal), investigation (equal), writing – review and editing (equal). **Jianrong Guo:** data curation (equal), formal analysis (equal), investigation (equal), writing – review and editing (equal). **Chao Ding:** conceptualization (supporting), methodology (supporting). **Yukai Jin:** conceptualization (supporting), methodology (supporting), writing – review and editing (supporting). **Haibo Qiu:** conceptualization (lead), methodology (lead), writing – review and editing (equal).

## Funding

This study was funded by the Natural Science Foundation of Guangdong Province (grant 2025A1515012334) and the Beijing CSCO Clinical Oncology Research Foundation (grant Y‐2019Roche‐157).

## Ethics Statement

The study was approved by the Institutional Review Board of Sun Yat‐sen. University Cancer Center (Guangzhou, China; Approval No: B2025‐146‐01), and was conducted in accordance with the 1964 Helsinki Declaration and its later amendments or comparable ethical standards. The requirement for informed consent was waived by the Institutional Review Board due to the retrospective nature of the study.

## Conflicts of Interest

The authors declare no conflicts of interest.

## Supporting information


**Data S1:** cam471619‐sup‐0001‐Supinfo.xlsx.

## Data Availability

The data that support the findings of this study are available on request from the corresponding author. The data are not publicly available because of privacy or ethical restrictions.
